# *Brucea javanica* oil alleviates intestinal mucosal injury induced by chemotherapeutic agent 5-fluorouracil in mice

**DOI:** 10.3389/fphar.2023.1136076

**Published:** 2023-02-20

**Authors:** Xinghan Zheng, Liting Mai, Ying Xu, Minghui Wu, Li Chen, Baoyi Chen, Ziren Su, Jiannan Chen, Hongying Chen, Zhengquan Lai, Youliang Xie

**Affiliations:** ^1^ School of Pharmaceutical Sciences, Guangzhou University of Chinese Medicine, Guangzhou, China; ^2^ Department of Pharmacy, Shenzhen University General Hospital/Shenzhen University Clinical Medical Academy, Shenzhen University, Shenzhen, Guangdong, China; ^3^ Pharmacy Department, Quanzhou Hospital of Traditional Chinese Medicine, Quanzhou, China; ^4^ Medical Insurance Office, Zhaoqing Hospital, Sun Yat-sen University, Zhaoqing, China; ^5^ Dongguan Institute of Guangzhou University of Chinese Medicine, Dongguan, China; ^6^ Guangzhou Baiyunshan Mingxing Pharmaceutical Co. Ltd, Guangzhou, China

**Keywords:** Brucea javanica oil, 5-fluorouracil, intestinal mucosal injury, Nrf2/HO-1, mucosal barrier

## Abstract

**Background:** *Brucea javanica* (L.) Merr, has a long history to be an anti-dysentery medicine for thousand of years, which is commonly called “Ya-Dan-Zi” in Chinese. The common liquid preparation of its seed, *B. javanica* oil (BJO) exerts anti-inflammatory action in gastrointestinal diseases and is popularly used as an antitumor adjuvant in Asia. However, there is no report that BJO has the potential to treat 5-Fluorouracil (5-FU)-induced chemotherapeutic intestinal mucosal injury (CIM).

**Aim of the study:** To test the hypothesis that BJO has potential intestinal protection on intestinal mucosal injury caused by 5-FU in mice and to explore the mechanisms.

**Materials and methods:** Kunming mice (half male and female), were randomly divided into six groups: normal group, 5-FU group (5-FU, 60 mg/kg), LO group (loperamide, 4.0 mg/kg), BJO group (0.125, 0.25, 0.50 g/kg). CIM was induced by intraperitoneal injection of 5-FU at a dose of 60 mg/kg/day for 5 days (from day 1 to day 5). BJO and LO were given orally 30 min prior to 5-FU administration for 7 days (from day 1 to day 7). The ameliorative effects of BJO were assessed by body weight, diarrhea assessment, and H&E staining of the intestine. Furthermore, the changes in oxidative stress level, inflammatory level, intestinal epithelial cell apoptosis, and proliferation, as well as the amount of intestinal tight junction proteins were evaluated. Finally, the involvements of the Nrf2/HO-1 pathway were tested by western blot.

**Results:** BJO effectively alleviated 5-FU-induced CIM, as represented by the improvement of body weight, diarrhea syndrome, and histopathological changes in the ileum. BJO not only attenuated oxidative stress by upregulating SOD and downregulating MDA in the serum, but also reduced the intestinal level of COX-2 and inflammatory cytokines, and repressed CXCL1/2 and NLRP3 inflammasome activation. Moreover, BJO ameliorated 5-FU-induced epithelial apoptosis as evidenced by the downregulation of Bax and caspase-3 and the upregulation of Bcl-2, but enhanced mucosal epithelial cell proliferation as implied by the increase of crypt-localized proliferating cell nuclear antigen (PCNA) level. Furthermore, BJO contributed to the mucosal barrier by raising the level of tight junction proteins (ZO-1, occludin, and claudin-1). Mechanistically, these anti-intestinal mucositis pharmacological effects of BJO were relevant for the activation of Nrf2/HO-1 in the intestinal tissues.

**Conclusion:** The present study provides new insights into the protective effects of BJO against CIM and suggests that BJO deserves to be applied as a potential therapeutic agent for the prevention of CIM.

## 1 Introduction

Up to right now, chemotherapy is still one of the first-line therapies for cancers. However, epidemiological studies reveal that practically 40% of cancer patients treated with a standard dose of chemotherapy develop clinical intestinal mucositis, which is referred to chemotherapeutic intestinal mucosal injury (CIM) and is characterized by severe diarrhea and inflammation (Thomsen and Vitetta, 2018; Cai et al., 2021). Chemotherapeutic agent 5-Fluorouracil (5-FU) plays an essential role in management of various cancers, and can cause CIM which threatens the effectiveness of therapy due to dose reduction and quality-of-life impairment among patients (Sonis et al., 2004). Since 5-FU-induced CIM exhibits stable, high incidences and similar pathological manifestations in mice, it is commonly used in animal models for CIM. The pathogenesis mechanisms of 5-FU-induced CIM include oxidative stress, inflammatory reaction, activation of apoptosis, interruption of proliferation in the intestinal epithelium, as well as destruction of intestinal mucosal barrier (Ribeiro et al., 2016). Currently, pharmacological interventions for CIM are still unavailable, except for the combined application of loperamide octreotide (LO), sulfasalazine, and probiotics for symptomatic relief and infection control (Keefe, 2007; Yeung et al., 2015; Bowen et al., 2019). Hence, it is in urgent need to develop safe and feasible therapeutic medications for the treatment of CIM. Recent research indicate that traditional Chinese medicine, such as patchouli oil (Gan et al., 2020), andrographolide (Xiang et al., 2020), berberine (Chen et al., 2020) and so on, show potent efficacy and safety in 5-FU-induced CIM in animal models, thus shedding new lights on the application of traditional Chinese medicine in the management of CIM.

*Brucea javanica* (L.) Merr, widely distributed in South China, has thousands years of history to be used for treating diarrhea and gastrointestinal diseases. (Yoon et al., 2020). The pharmacologically active composition of *B. javanica* is referred to as *Brucea javanica* oil (BJO). It is generally considered to be the fatty oil extraction from the dried ripe fruit of *Brucea javanica* by petroleum ether or hexane. BJO contains multiple effective ingredients, including quassinoids and fatty acids such as oleic acid and linoleic acid (Chen et al., 2013; Yan et al., 2015). In our former studies, BJO and its active components demonstrated antioxidant and anti-inflammatory properties and maintained intestinal barrier integrity, contributing to amelioration of ulcerative colitis and inflammatory bowel diseases in mice (Huang et al., 2017; Huang et al., 2019). Additionally, BJO treatment is safe and has fewer side effects and thus widely used as an effective assistant treatment of various malignant tumor patients during radiotherapy and chemotherapy (Xu et al., 2016; Thomsen and Vitetta, 2018).

Taken into considerations that CIM shares similar symptoms and pathological mechanisms with inflammatory bowel diseases and dysentery (Hamouda et al., 2017; Rtibi et al., 2018), the present work was focused on the potential effects of BJO in the treatment of CIM in a 5-FU-induced mice model. Additionally, the protective effects of BJO against oxidative stress, inflammation, apoptosis of intestinal epithelium and intestinal barrier disruption were investigated to explore the pharmacological mechanisms of BJO.

## 2 Materials and methods

### 2.1 Reagents

BJO was provided by Ming Xing Pharmaceutical Co. Ltd. (Guangzhou, Guangdong, China) with the number (Lot: 20180902), stored in 4°C refrigerator of room A204, laboratory of the School of Pharmaceutical Sciences, Guangzhou University of Chinese Medicine, Guangzhou, China. To obtain the emulsion, BJO was prepared as previously reported (Wang et al., 2020). We mixed the appropriate amount of soybean lecithin and water to obtain the blank emulsion. After mixing the proper amount of BJO with the blank emulsion for 9 min, we obtained the milky white oil emulsion like cream with high-pressure homogenization. All processes were followed the standard of the ministry of Health of the people’s Republic of China (WS3-B-3646-98).

5-FU was purchased from Sigma Corporation (United States). LO was produced by Yang Senlin Pharmaceutical (Xi’an, China). Malondialdehyde (MDA) and superoxide dismutase (SOD) assay kits were bought from Jiancheng Biotechnology Company (Nanjing, Jiangsu, China). The ELISA (enzyme-linked-immunosorbent serologic assay) kits (tumor necrosis factor-α (TNF-α), interleukin-6 (IL-6), interleukin-4 (IL-4), interleukin-1β (IL-1β), inducible nitric oxide synthase (iNOS), and diamine oxidase (DAO)) were obtained from Shanghai MLBIO Biotechnology (Shanghai, China). Primary antibodies against proliferating cell nuclear antigen (PCNA), cyclooxygenase-2 (COX-2), zonula occludens-1 (ZO-1), occludin, claudin-1, and β-actin were purchased from Affinity Biosciences (OH, United States). TRIzol^®^ reagent was provided by Thermo Fisher (Waltham, MA, United States). Mice primers for occludin, claudin-1, CXC chemokine ligand 1 (CXCL1) and CXC chemokine ligand 2 (CXCL2) were purchased from (Sangon Biotech, Shanghai, China). Other required materials were bought from Vazyme Biotech (Nanjing, China).

### 2.2 Animals

The experimental animals were Kunming mice (weighting 22–25 g, half male and female) provided by the Laboratory Animal Centre, Guangzhou University of Chinese Medicine, Guangzhou, China (approval number: 44007200079662). Mice were cared under the standard laboratory conditions, which is temperature 22°C ± 2°C, humidity 50% ± 10%, and 12 h dark-light cycle. And they were allowed to freely consume sterilized water and standard chow. All experimental procedures were approved by the Animal Ethics Committee of Guangzhou University of Chinese Medicine.

### 2.3 Induction of CIM by 5-FU and treatments

Kunming mice were randomly assigned into six groups (n = 10): normal group; 5-FU group (5-FU, 60 mg/kg/day for 5 days, intraperitoneal injections); LO group (loperamide; 4 mg/kg/day for 7 days, oral administration); BJO group (0.125 (BJOL), 0.250 (BJOM), 0.500 (BJOH) g/kg/day for 7 days, oral administration). According to previous research (Zheng et al., 2019), 5-FU intraperitoneal injection (from day 1 to day 5); 30 min later, oral administration of BJO and LO was carried out (from day 1to day 7). The normal group mice were injected with physiological saline and orally received blank emulsion. The 5-FU group mice were injected with 5-FU and orally given blank emulsion. Body weight, stool consistency, food intake, and general appearance of mice were recorded daily. Diarrhea was measured using the mean scores. As shown in Table 1, the severity was quantified according to a previously described procedure (Huang et al., 2009). After the 7-day treatment, the blood was obtained after enucleation of eyeball, and mice were sacrificed by cervical dislocation. After 2 h, blood was centrifuged for 15 min at 3500 rpm and 4°C in refrigerate centrifuge, and serum was collected.

**TABLE 1:** Criteria for scoring diarrhea.

Score	Feature
0-normal	normal stool or the absence of stool
1-slight	slightly wet and soft stool
2-moderate	wet and unformed stool with moderate perianal staining of the coat
3-severe	watery stool with severe perianal staining of the coat

### 2.4 Histopathological evaluation of intestine

After the mice were sacrificed, the ileum tissue was removed immediately and put into the fixation solution (4% paraformaldehyde) for 24 h, then the tissue was immersed in wax, and sections (5 μm thick) were dewaxed and stained as recommended by the standard operating protocols. The macroscopic injuries of H&E staining segments of the ileum were assessed after microscopical observation.

### 2.5 Immunohistochemical analysis

The ileum tissue was dewaxed and hydrated by paraffin-embedded slices, then placed in citrate antigen repair buffer (pH 6.0) for antigen repair. After that, the tissue endogenous peroxidase was blocked and 3% BSA was closed. According to experimental conditions, the primary and secondary antibodies were incubated successively. Finally, the section was colored by DAB (diaminobenzidine method) and the hematoxylin stained cell nucleus for 3 min. Immunohistochemical staining was used to determine either PCNA or COX-2, and the results were analyzed by using the graphic analysis system. The immunohistochemical images were captured by a microscope.

### 2.6 Cytokines analysis by commercial assay kits and ELISA

The SOD and MDA assay was performed in accordance with the instructions of commercial assay kits. At a low temperature, the samples were homogenized with appropriate amount of pre-cold PBS and centrifuged (4000 rpm/min, 20 min), and then collect the supernatant. The content of proinflammatory factors (TNF-α, IL-6, IL-4, IL-1β, iNOS) and DAO were measured by ELISA kits in the light of the manufacturer’s instruction.

### 2.7 RT-polymerase chain reaction

Total RNA was collected from mice intestine tissues using TRIzol^®^ reagent. 1 μg RNA was reverse transcribed to produce cDNA using the HiScript™ cDNA Synthesis Kit. Then amplification program was done in line with the protocols. Primer sequences for claudin-1, occludin, CXCL1, CXCL2, NLRP3, and GAPDH are stated in Table 2.

**TABLE 2:** Real-time PCR primer sequences.

Gene		Primer sequences (5′-3′)
CXCL1	FORWARD	ATG​GCT​GGG​ATT​CAC​CTC​AAG​AAC
	REVERSE	AGT​GTG​GCT​ATG​ACT​TCG​GTT​TGG
claudin-1	FORWARD	GCT​GGG​TTT​CAT​CCT​GGC​TTC​TC
	REVERSE	CCT​GAG​CGG​TCA​CGA​TGT​TGT​C
Occludin	FORWARD	TGG​CTA​TGG​AGG​CGG​CTA​TGG
	REVERSE	AAG​GAA​GCG​ATG​AAG​CAG​AAG​GC
CXCL2	FORWARD	CAC​TGG​TCC​TGC​TGC​TGC​TG
	REVERSE	GCG​TCA​CAC​TCA​AGC​TCT​GGA​TG

### 2.8 Western blot

The total protein was isolated and extracted from intestine tissues using cold RIPA lysis buffer supplemented with protease inhibitor, and then determined and denatured. Equal amounts of the protein samples were subjected to SDS-PAGE and transferred onto PVDF membranes. The non-specific binding sites were blocked with 5% skimmed milk at 25°C, and then the membranes cleaned in TBST for 3 times. After that, the blocked membranes were incubated at 4°C overnight with the indicated primary antibodies, and then treated with HRP-conjugated secondary antibody for 2 h at room temperature. Protein bands were visualized using ECL luminescence solution in the detection system and quantified with ImageJ software.

### 2.9 Data analysis

All values were represented as means ± standard error (SEM) except for data that did not obey normal distribution, which were expressed as M (*P*_25_∼*P*_75_). SPSS 23.0 software (SPSS Inc., Chicago, IL, United States) was used to accomplish the statistical analyses. Data processing was performed by one-way analysis of variance (ANOVA), followed by Tukey’s *post hoc* test to perform multiple comparison procedures. Significant differences between groups were reflected as *p* < 0.05.

## 3 Results

### 3.1 BJO improved 5-FU-induced intestinal mucositis

**FIGURE 1:**
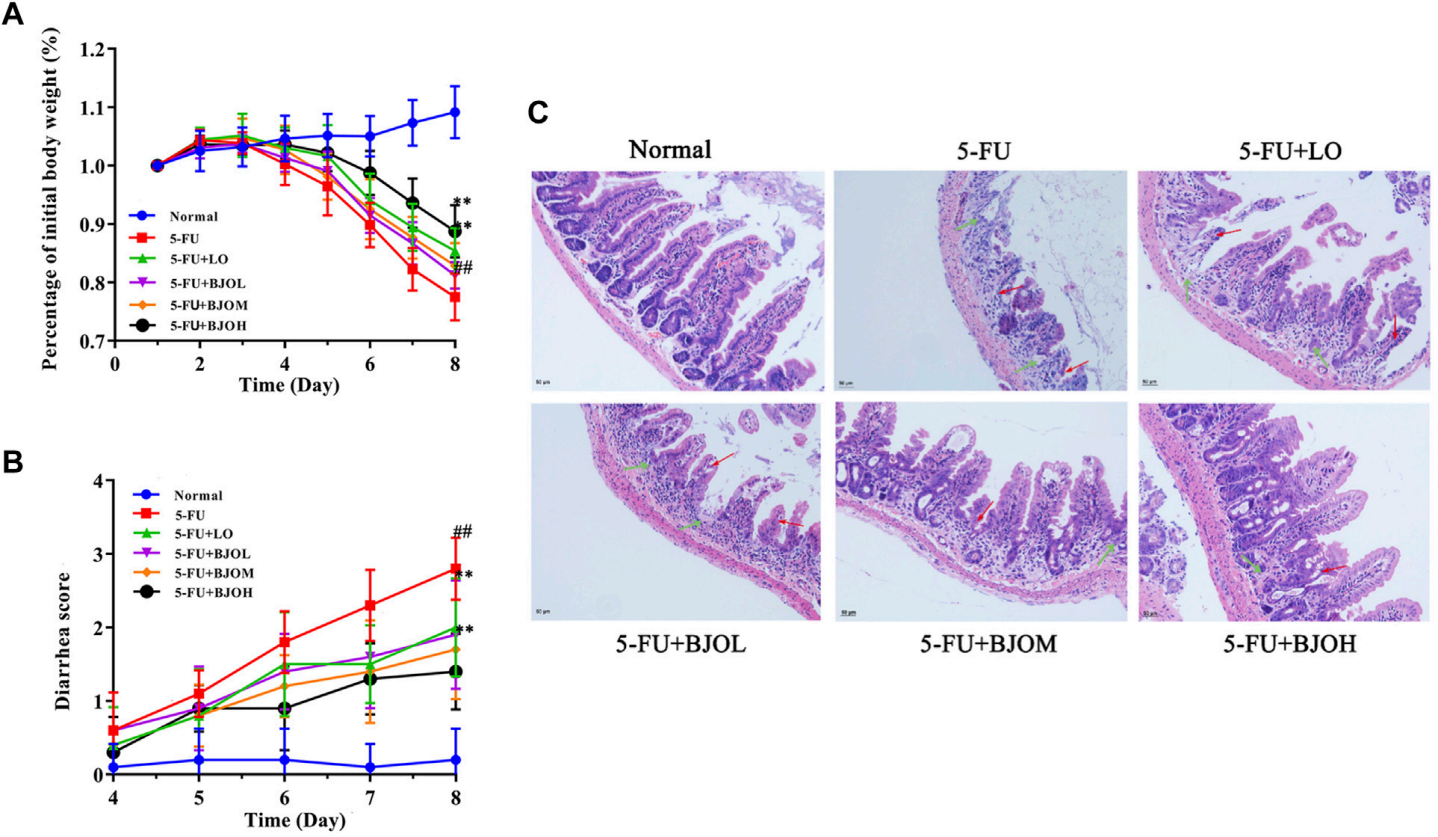
BJO improved 5-FU-induced intestinal mucositis. **(A)** Percentage of initial body weight (%, *n* = 10) **(B)** Diarrhea score (*n* = 10). **(C)** The ileum histopathology of CIM mice (200×, *n* = 6). Red arrow: shortened intestinal villus; green arrow: loss of crypt architecture. **p < 0.01 compared with 5-FU group; ^##^ *p* < 0.01 compared with normal group.

The clinical symptoms of 5-FU-induced CIM generally included weight loss, abdominal pain, and severe diarrhea (Atiq et al., 2019; Xiang et al., 2020). In this experiment, the potential effect of BJO treatment on 5-FU-induced CIM was investigated through assessment of body weight, diarrhea status, and histological changes in the ileum. As illustrated in Figure 1A, the mice’s weight showed a gradual decrease from the fourth day of 5-FU injection. Oral medication of 0.500 g/kg BJO or the positive control drug LO significantly ameliorated weight loss induced by 5-FU (*p* < 0.01) (Figure 1A). Similarly, extensive diarrhea resulted from the 5-FU injection was alleviated by administration of BJO in a concentration-dependent manner (*p* < 0.01) (Figure 1B). The H&E staining demonstrated mucosal erosion, disruption of crypt-villus structures, and subacute inflammation in the ileum of mice in the 5-FU-treated groups (Figure 1C). However, BJO treatment significantly reversed the damage of mucosal epithelium and subacute inflammation, as implied by the recovery of mucosa thickness, villus height, crypt depth, and prevention of inflammatory cells infiltration (Figure 1C). Altogether, these results suggested that BJO treatment ameliorates intestinal mucositis caused by 5-FU.

### 3.2 BJO repressed oxidative stress induced by 5-FU

**FIGURE 2:**
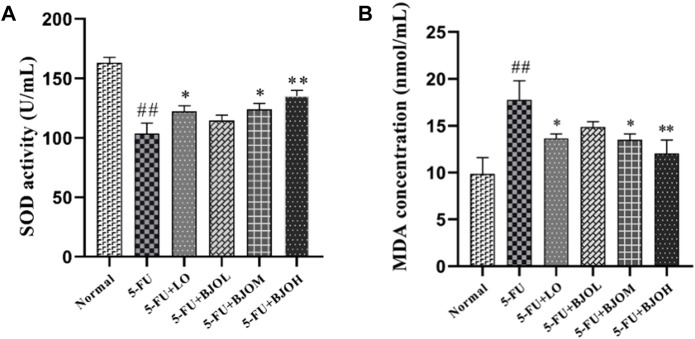
The effect of BJO in markers of oxidative stress in serum (*n* = 8). **(A)** SOD activity, **(B)** MDA concentration. *p < 0.05, **p < 0.01 compared with 5-FU group; ^##^ *p* < 0.01 compared with normal group.

**FIGURE 3:**
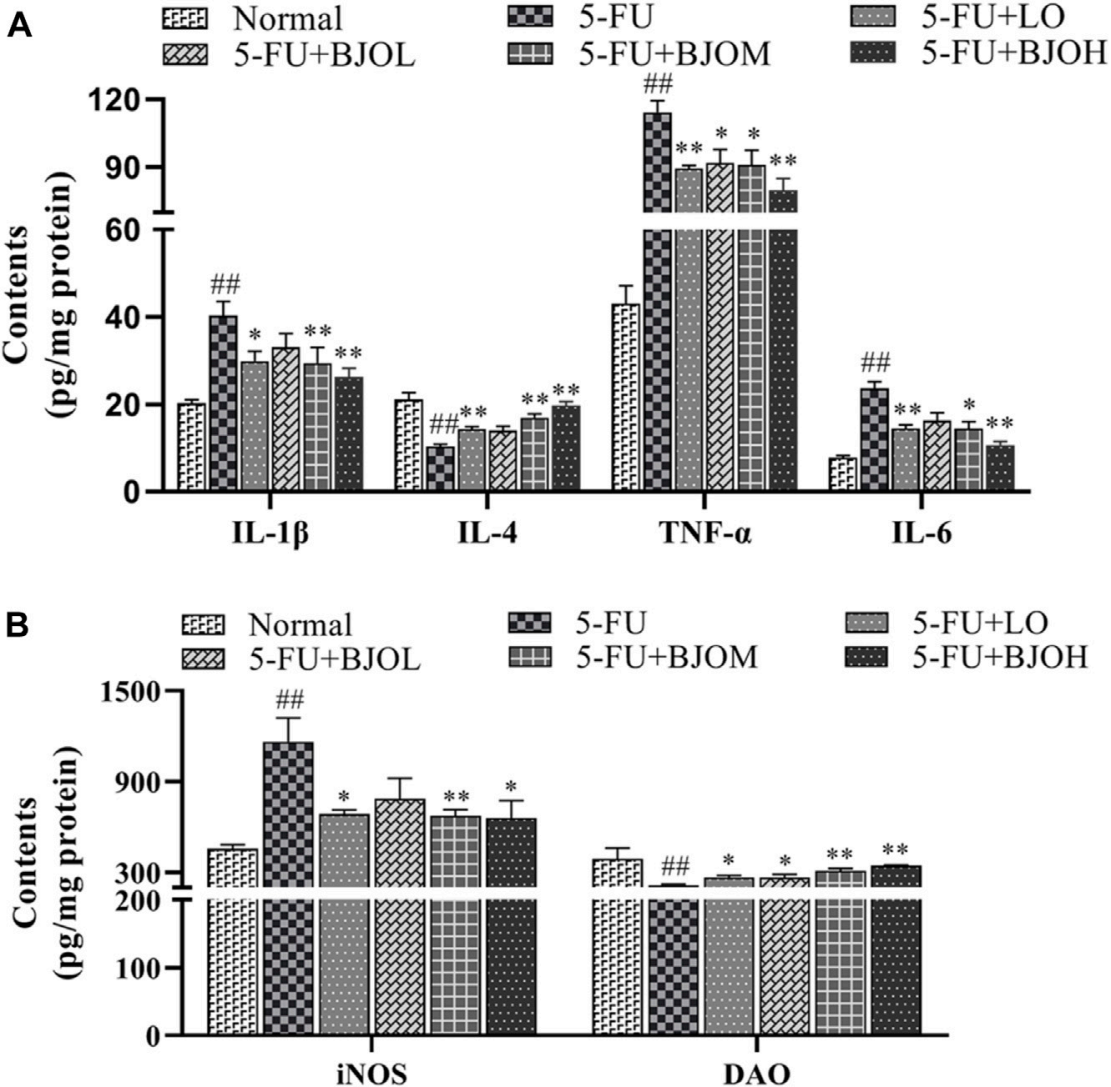
The effect of BJO on inflammatory cytokines and intestinal permeability in intestine tissue of CIM mice. **(A)**The levers of IL-1β, IL-4, TNF-α and IL-6 in intestinal tissue. **(B)** The levers of iNOS and DAO in intestinal homogenates. *p < 0.05, **p < 0.01 compared with 5-FU group; ^##^ *p* < 0.01 compared with normal group.

We evaluated whether BJO decreased 5-FU-induced oxidative stress in the serum. SOD exerts an important influence in maintaining the oxidation and antioxidant balance of the body (Cao et al., 2018). The lipid peroxidation results in the accumulation of MDA, and then ultimately leads to disturbance of bio-membranes’ fluidity and increase of permeability of bio-membranes (Cui et al., 2018). As shown in Figure 3, there was a significant depletion of serum SOD activity accompanied by an elevation of serum MDA levels in the 5-FU-treated mice compared to that in the normal control (*p* < 0.01). Following treatment with BJO (0.250 g/kg, 0.500 g/kg), SOD activity significantly rebounded (*p* < 0.05, *p* < 0.01), and MDA content was reduced (*p* < 0.05, *p* < 0.01) (Figure 2). Thus, our observations show that BJO has antioxidant capacity and contributes to elimination of oxidative stress in 5-FU-induced CIM.

### 3.3 BJO suppressed 5-FU-triggered intestinal inflammation

To further investigate the impact of BJO against 5-FU-induced inflammation in intestine tissue, the expression levels of intestinal inflammatory cytokines were tested. As shown in Figure 3A, compared with normal group, the pro-inflammatory factors IL-1β, TNF-α, and IL-6 of 5-FU group were significantly raised (all *p* < 0.01), however, administration of BJO led to an evident dose-dependent reduction in 5-FU-induced elevation of pro-inflammatory factors IL-1β (*p* > 0.05, *p* < 0.05, *p* < 0.01, respectively), TNF-α (*p* < 0.05, *p* < 0.05, *p* < 0.01, respectively), and IL-6 (*p* > 0.05, *p* < 0.05, *p* < 0.01, respectively). For the release of anti-inflammatory IL-4, BJO shown an obvious dose-dependent increase (*p* > 0.05, *p* < 0.01, *p* < 0.01, respectively).

Furthermore, the intestinal levels of iNOS and DAO were investigated. The inflammatory marker iNOS was significantly upregulated after 5-FU treatment (*p* < 0.01), but it was reversed by BJO (*p* > 0.05, *p* < 0.01, *p* < 0.05, respectively) (Figure 3B). DAO is especially active in the intestinal mucosa and regulates the rapidly proliferating intestinal mucosa (Fukuda et al., 2019). DAO can penetrate across the intestinal barrier and shuttle to the circulation when the mucosal barrier is disrupted, so a decrease of intestinal DAO is obvious during intestinal mucositis. The activity of DAO was repressed by 5-FU (*p* < 0.01), but activity was recovered following BJO administration (*p* < 0.05, *p* < 0.01, *p* < 0.01, respectively) (Figure 3B).

**FIGURE 4:**
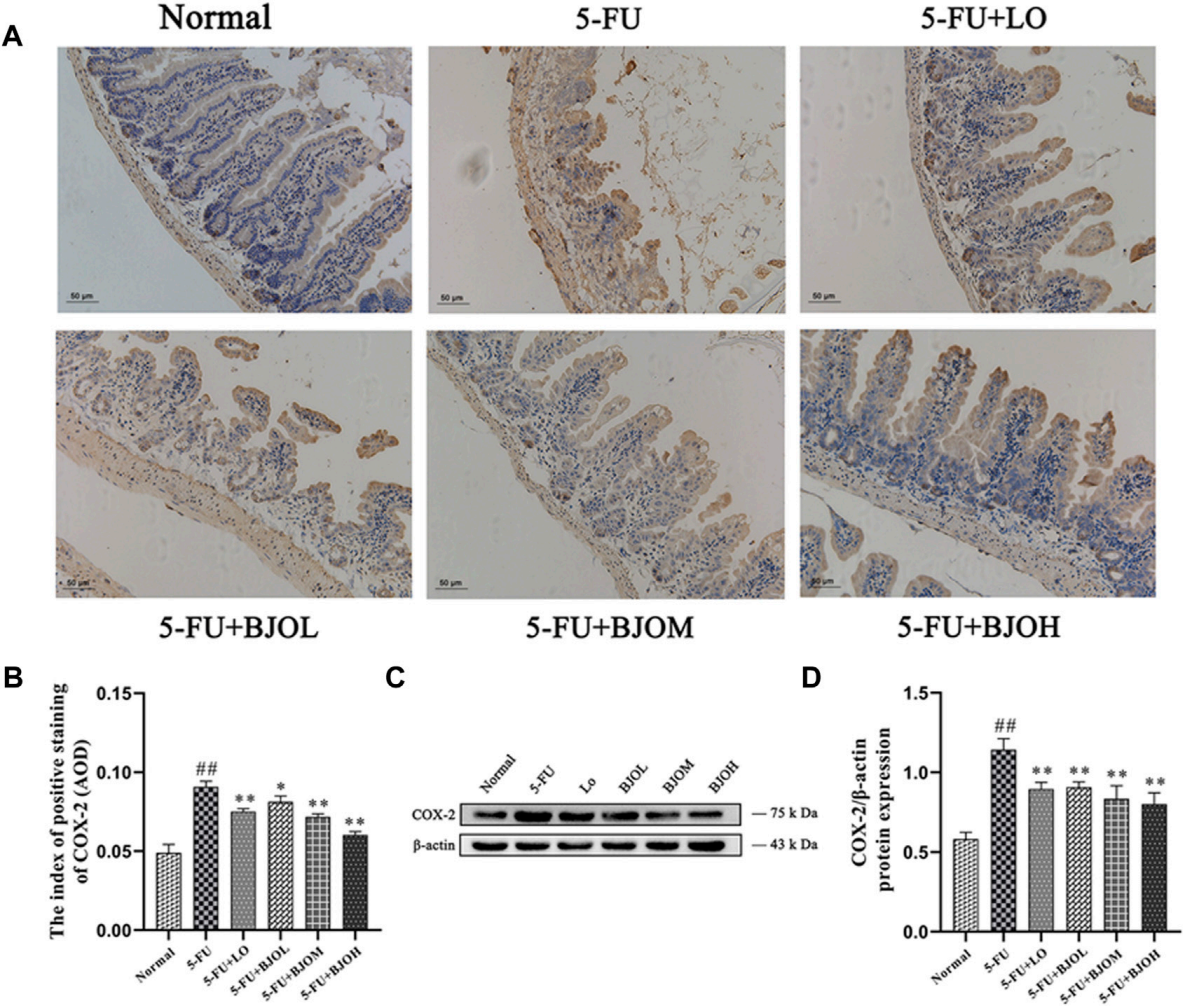
The effects of BJO on the expression of COX-2. **(A, B)** The expression of COX-2 (A, 200×) was detected by immunohistochemical staining in ileum sections (*n* = 3). **(C, D)** The expression of COX-2 was detected by Western blot (*n* = 3). *p < 0.05, **p < 0.01 compared with 5-FU group; ^##^ *p* < 0.01 compared with normal group.

Moreover, compared with the 5-FU group, the IHC staining and Western blot data of BJO shown a downward trend (*p* < 0.05, *p* < 0.01, *p* < 0.01; *p* < 0.01, *p* < 0.01, *p* < 0.01, respectively), indicating that the 5-FU-induced increase in COX-2 was attenuated by BJO (Figure 4).

**FIGURE 5:**
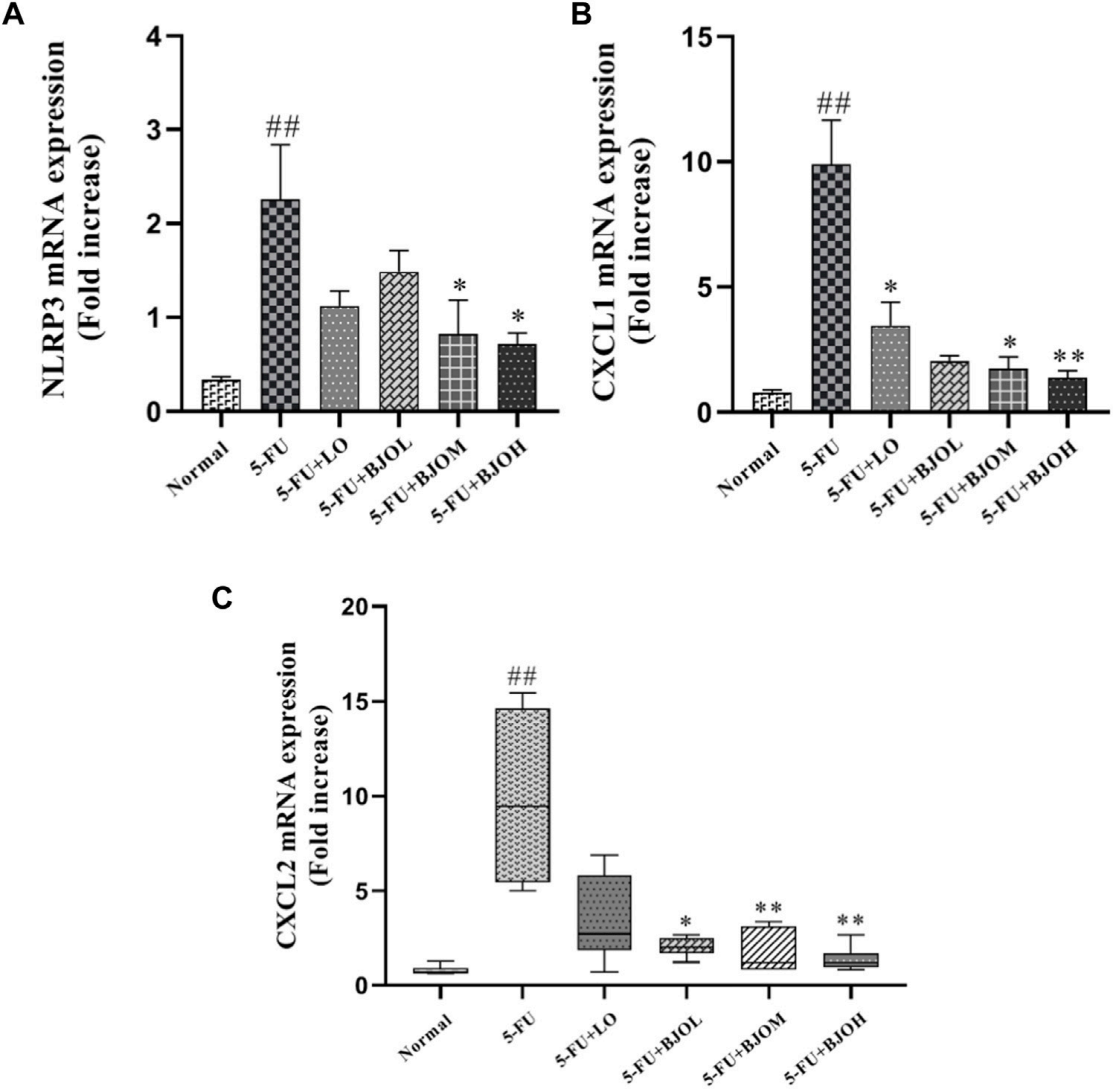
The effects of BJO on the transcription levels of NLRP3 and chemokines in intestinal tissues of CIM mice (*n* = 6). **(A)** NLTP3 mRNA expression; **(B)** CXCL1 mRNA expression; **(C)** CXCL2 mRNA expression. *p < 0.05, **p < 0.01 compared with 5-FU group; ^##^ *p* < 0.01 compared with normal group.

CXCL1 and CXCL2 are the potent neutrophils chemoattractants that mediate intestinal inflammatory responses and are related to the pathogenesis of CIM (Guo et al., 2020; Hu et al., 2021). We detected the mRNA amounts of CXCL1 and CXCL2 in intestinal tissue. Both of their productions were enhanced by 5-FU (*p* < 0.01), while BJO significantly reduced the productions of them (*p* > 0.05, *p* < 0.05, *p* < 0.01; *p* < 0.05, *p* < 0.01, *p* < 0.01, respectively) (Figures 5B, C). Moreover, Chemokines CXCL1 and CXCL2 are responsible for the inflammasome activation, and release IL-1β in macrophages (Boro and Balaji, 2017). We observed that compared with the normal group, mRNA expression of NLRP3 was increased more than three-fold in the intestine of the 5-FU group. In all dose, BJO suppressed NLRP3 expression in intestinal tissue (*p* > 0.05, *p* < 0.05, *p* < 0.05, respectively) (Figure 5A).

Therefore, these findings support the conclusion that BJO exerts anti-inflammatory properties against 5-FU-induced CIM.

### 3.4 BJO inhibited 5-FU-triggered epithelial apoptosis and proliferation inhibition

**FIGURE 6:**
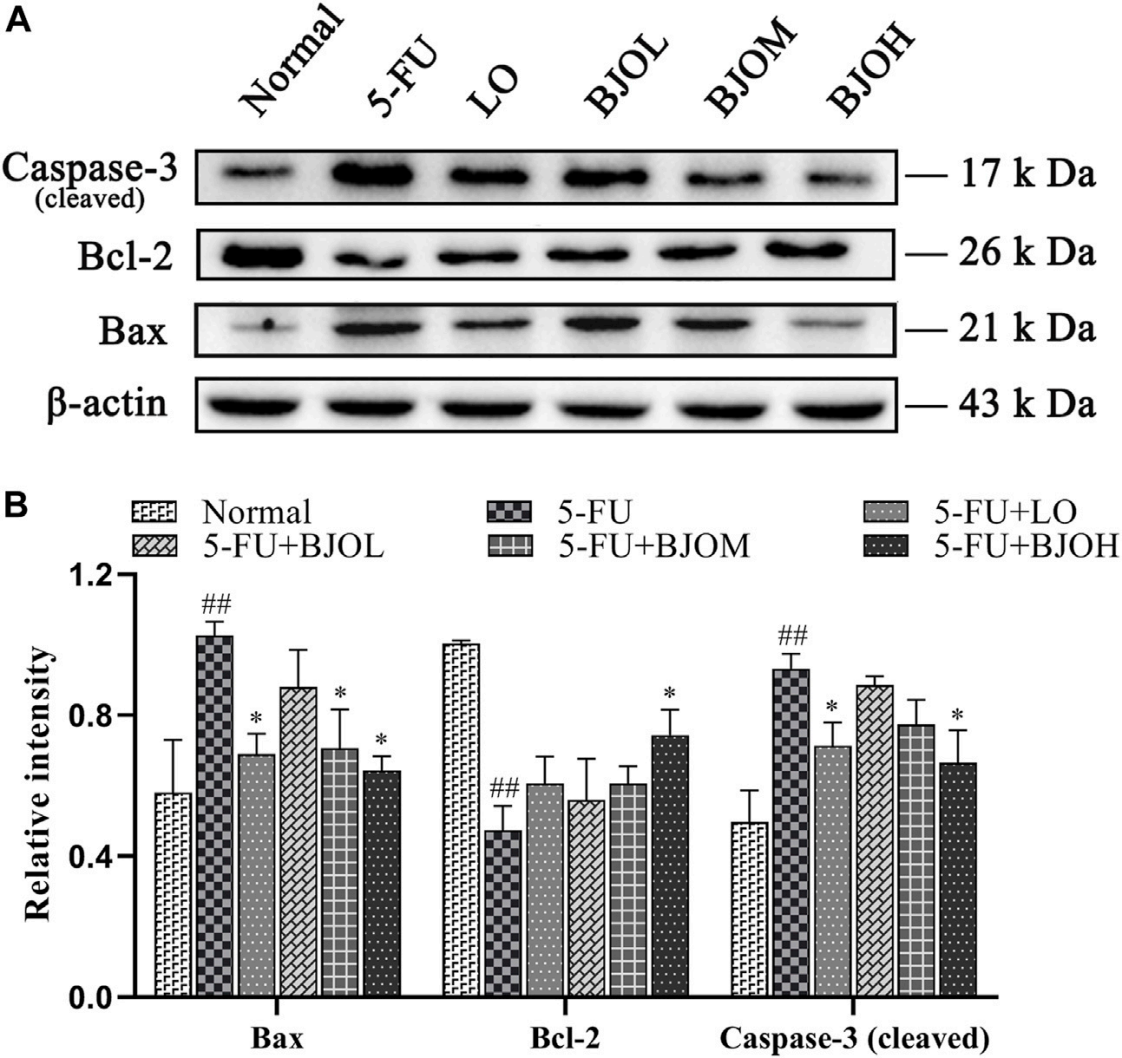
The effects of BJO on the protein expression of Bax、Bcl-2 and caspase-3 (cleaved) in intestinal tissues in CIM mice. **(A)** The protein bands of Bax、Bcl-2 and caspase-3 (cleaved); **(B)** The statistical analysis of Bax、Bcl-2 and caspase-3 (cleaved) protein expressions (*n* = 3). *p < 0.05, **p < 0.01 compared with 5-Fu group and ^##^ *p* < 0.01 compared with normal group.

Activation of apoptosis and proliferation inhibition of crypt cells in the intestinal epithelium is one of the key links to CIM (De Angelis et al., 2006). To explore whether BJO could exert an anti-apoptotic effect in 5-FU-induced CIM, the apoptosis-related molecules’ expressions were quantified. As shown in Figure 6, the 5-FU group exhibited significant augmentation on the protein level of Bax and cleaved-caspase-3 (all *p* < 0.01), together with a diminution of Bcl-2 expression (*p* < 0.01), compared with the normal group, indicating that 5-FU facilitated cell apoptosis. Treatment of BJO (0.500 g/kg) remarkably inhibited 5-FU- stimulated cell apoptosis in CIM mice (all *p* < 0.05).

**FIGURE 7:**
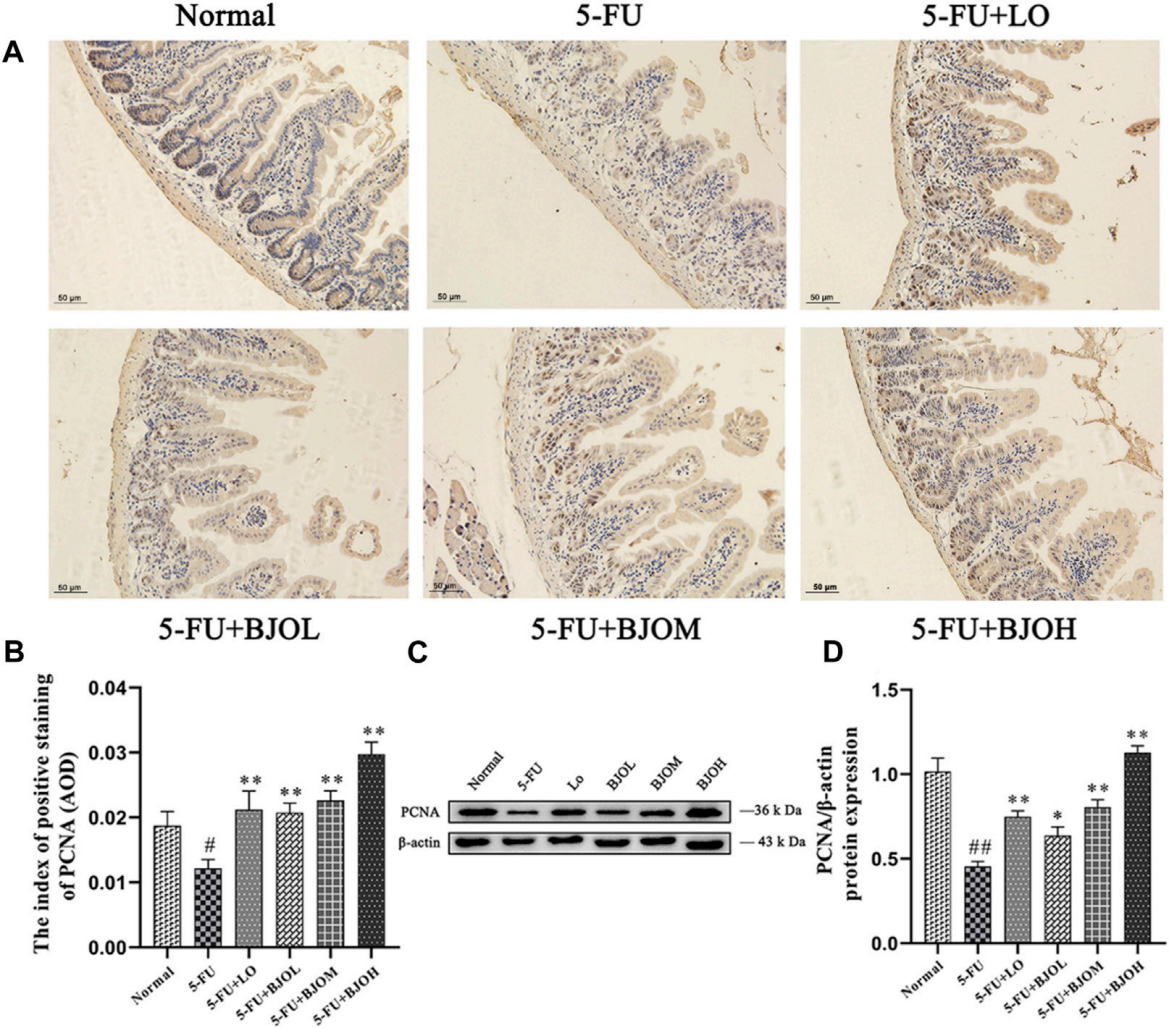
The effects of BJO on the expression of PCNA in ileum sections. **(A, B)** The expression of PCNA was detected by immunohistochemical staining (200×, *n* = 3). **(C, D)** The expression of PCNA was detected by western bolt (*n* = 3). *p < 0.05, **p < 0.01 compared with 5-FU group; ^##^ *p* < 0.01 compared with normal group.

The cell-proliferation capability of enterocyte was evaluated by determining the PCNA expression using immunohistochemistry assay and Western blotting. Treatment with 5-FU at 60 mg/kg dose hampered PCNA expression compared to normal control group (all *p* < 0.01). By contrast, BJO concentration-dependent increased the expression of PCNA (*p* < 0.05, *p* < 0.01, *p* < 0.01, Figures 7C, D), especially the crypt-localized PCNA (all *p* < 0.01, Figures 7A, B). These data suggest that BJO is able to enhance the survival of crypt cells following chemotherapy and assist in the recovery of the impaired mucosal membrane.

### 3.5 BJO alleviates 5-FU induced disruption of tight junction proteins

**FIGURE 8:**
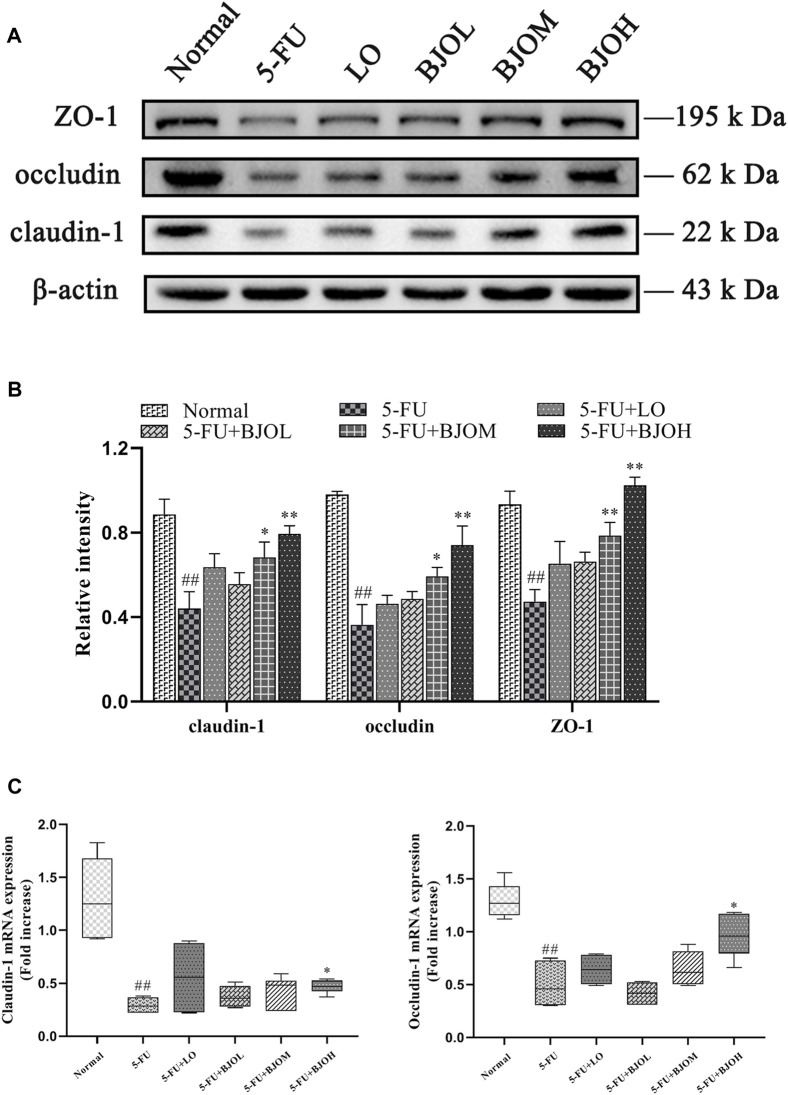
The effects of BJO on the expression of ZO-1, occludin, and claudin-1 in intestinal tissues of CIM mice. **(A)** The protein bands of ZO-1, occludin, and claudin-1;**(B)** The statistical analysis of ZO-1, occludin, and claudin-1 protein expressions (*n* = 3); **(C)**The mRNA expressions of claudin-1 and occludin (*n* = 6). *p < 0.05, **p < 0.01 compared with 5-FU group; ^##^ *p* < 0.01 compared with normal group.

Destruction of the mucosal barrier induced by chemotherapy might cause mucosal barrier dysfunction (Sanchez de Medina et al., 2014). We examined the key indicator of the tight junction markers, including ZO-1, occludin, and claudin-1, which are closely associated with the epithelial cell permeability and mucosal barrier function. As shown in Figures 8A, C, the tight junction protein expressions of ZO-1, occludin, and claudin-1 were markedly decreased in mice stimulated with 5-FU (all *p* < 0.01), but they were obviously restored by BJO with 0.500 g/kg (all *p* < 0.01). By contrast, the routine anti-diarrheal drug loperamide did not show an ameliorative effect on their expressions (all *p* > 0.05, Figures 8A, B). As shown in Figure 8C, the mRNA expressions of occludin and claudin-1 were markedly decreased in mice stimulated with 5-FU (all *p* < 0.01), while they were significantly increased in BJO (0.500 g/kg, all *p* < 0.05) Thus, these data suggested that BJO could sustain the intestinal mucosa barrier function and recover from damage caused by 5-FU.

### 3.6 BJO alleviated CIM by activating Nrf2/HO-1 signaling pathway

**FIGURE 9:**
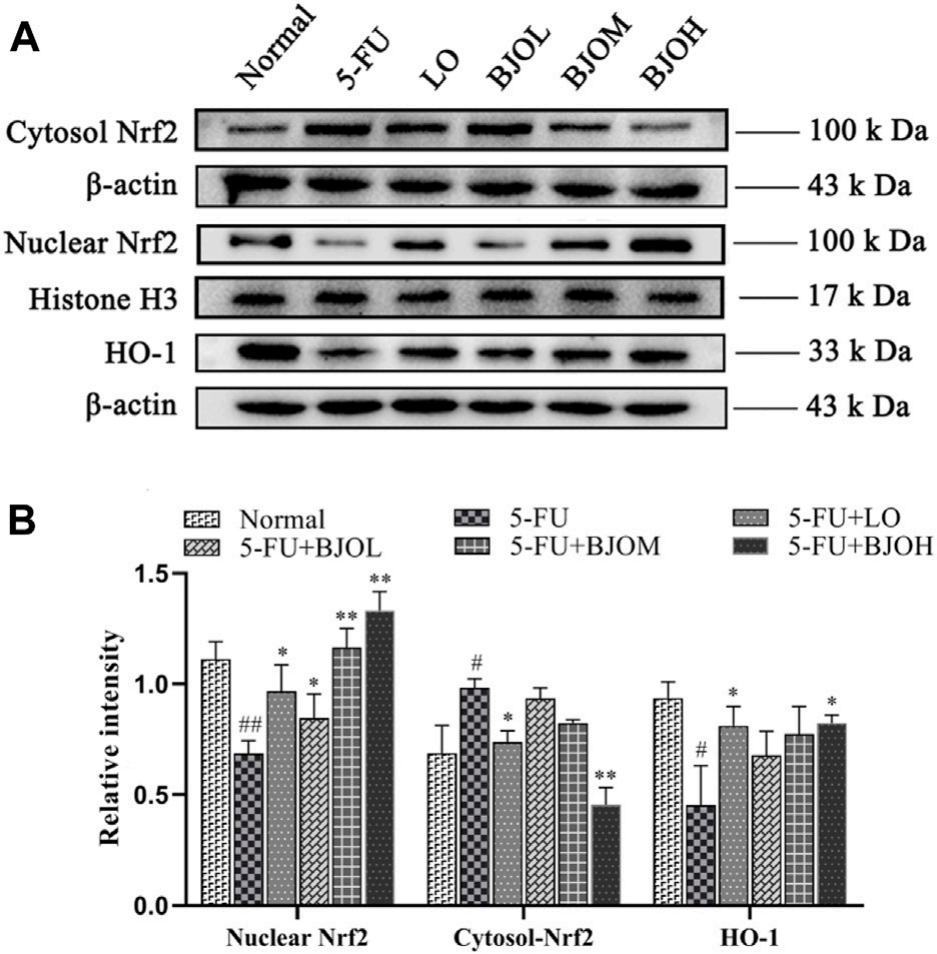
The effects of BJO on the signaling pathways of Nrf2/HO-1 involved in CIM mice. **(A)** The related protein bands of Nrf2/HO-1 signaling pathways; **(B)** The statistical analysis of Nrf2/HO-1 signaling pathways (*n* = 3). *p < 0.05, **p < 0.01 compared with 5-FU group; ^##^ *p* < 0.01 compared with normal group.

We further explored the mechanisms underlying the antioxidant and anti-inflammatory effects of BJO by investigating Nrf2/HO-1 signaling pathway. As shown in Figure 9, we observed a significant elevation in the cytoplasmic content of Nrf2, accompanied by a reduction of nuclear content of Nrf2 and the downstream target protein HO-1 (*p* < 0.05, *p* < 0.01, *p* < 0.05, respectively), in the 5-FU group, suggesting that Nrf2 is inactivated during CIM. However, BJO significantly facilitated the nuclear potion of Nrf2 (*p* < 0.05, *p* < 0.01, *p* < 0.01, respectively) and the followed transcription of HO-1(*p* > 0.05, *p* > 0.05, *p* > 0.05, respectively), thus indicating that BJO is able to activate the anti-oxidant Nrf2/HO-1 signaling pathway (Figure 9).

Consistent with the above observations, our results indicated that the antioxidant and anti-inflammatory effects of BJO are associated with activating Nrf2/HO-1 signaling pathway.

## 4 Discussion

BJO emulsion injection has been extensively applied as adjunctive therapies in combination with radiochemotherapy for colorectal carcinoma, gastric cancer, liver cancer, and other diseases (Ye et al., 2016; Chen and Wang, 2018; Wang et al., 2020). BJO exerts synergetic antitumor effects by increasing the sensitivity of tumor cells to chemotherapeutic agents, reversing drug resistance, maintaining the quality of life, and decreasing the frequency of adverse reactions during treatment (Ye et al., 2016; Chen and Wang, 2018; Wang et al., 2020). Our study extends cognizance to the therapeutic effects of BJO in chemotherapy-induced intestinal mucositis, considering that BJO treatment prevented weight loss, ameliorated diarrhea syndrome, and recovered mucosal epithelium impairment in a 5-FU-stimulated CIM mice model. These mucosal protective effects of BJO were attributable to its antioxidant and anti-inflammatory properties, anti-apoptosis and proliferation activation in mucosal epithelial cells, and improvement of mucosa barrier function. Indeed, these findings are consistent with our former observations that BJO has the therapeutic potential for treating intestinal diseases (Huang et al., 2017; Yoon et al., 2020), and that BJO can help reduce the adverse effects of radiochemotherapy, such as abdominal pain, diarrhea, and myelotoxicity in clinics (Xu et al., 2016). Thus, the present findings may broaden our understanding of the clinical application of BJO in treatment of chemotherapy-induced intestinal mucositis.

Mucositis contains complex biological mechanisms that occur in five stages: initiation, primary damage reaction, signal multiplication, ulceration, and recovery (Sonis, 2004). The increasing ROS and the antioxidant defense mechanisms‘ damage are considered to be causative mediators in the initial stage of CIM (Keefe, 2007). Indeed, our results demonstrated that the serum activity of SOD was reduced, but that of MDA was elevated in 5-FU-treated mice. BJO treatment could reverse the changes of SOD and MDA content, thus confirming that BJO could prevent oxidative stress and protect against CIM at the initial stage.

Excessive reactive oxygen species (ROS) generation in mucosal cells further induces negative events, including inflammatory and immune responses, which have detrimental effects on intestinal epithelial cells, subsequently initiating inflammatory signaling cascades and leading to intestinal disorder (Ribeiro et al., 2016). Our results suggested that BJO had an anti-inflammatory effects in the following ways: (1) BJO repressed the secretion of proinflammatory factors in the mucosa, including TNF-α, IL-1β and IL-6; (2) BJO decreased the expression of COX-2, which is the rate-limiting enzyme for the process of prostanoids’ synthesis and is irregularly upregulated during pathogenesis of intestinal inflammation (Lu and Zhu, 2014); (3) BJO suppressed the release of the chemical attractors CXCL1/CXCL2, which mediate the neutrophil recruitment, an essential preliminary action of tissue inflammation or repair (De Filippo et al., 2013); and (4) BJO restrained the activation of NLRP3 inflammasome, which drives host and immune responses by releasing cytokines and alarmins into circulation and has been revealed to be the vital contributor in the pathogenesis of various diseases involving inflammation (He et al., 2019; Li et al., 2020; Xu et al., 2022). In fact, these inflammatory cascades are not separated because proinflammatory cytokines might amplify the primary inflammatory signal, resulting in the transcription of COX-2 (Sonis, 2004), and the release of the chemical attractor CXCL1/CXCL2 may participate in NLRP3 activation (Serdar et al., 2020). Thus, it is most likely that BJO exerts synergetic effect on these inflammatory cascades.

Chemotherapy-induced intestinal mucositis cause a significant elevation in intestinal epithelial cells apoptosis, subsequently resulting in mucosal damage (Keefe et al., 2000). According to the present observations, BJO downregulated cleaved-caspase-3 and the pro-apoptotic Bax while augmented the anti-apoptotic Bcl-2, thus suggesting that BJO could alleviate the apoptotic process in CIM and might prevent atrophy of the villus and injury to the enterocytes in the intestine (Boeing et al., 2021).

Additionally, BJO upregulated crypt-localized PCNA, a major endogenous marker of cell proliferation capability (Phoophitphong et al., 2012), indicated that BJO could enhance proliferation of intestinal epithelial cells. Taken together, these findings demonstrated that BJO maintains the balance between apoptosis and proliferation of intestinal epithelial cells, finally recovering chemotherapy-induced mucosal damage.

Furthermore, disruption of mucosal barrier function is accompanied by oxidative stress, excessive inflammatory, and extensive apoptosis of epithelial cells (Turner, 2009). The tight junctions are multi-protein complexes that can constitute paracellular barriers to maintain the homeostasis of mucosal. In this experiment, the pharmacological action of BJO on mucosal barrier function was evaluated by detecting the levels of ZO-1, occludin, and claudin-1. Among these proteins, the claudin family reflects tight junction permeability (Tsukita et al., 2019). Peripheral membrane proteins like ZO-1, presents to be contributory to tight junction assembly and maintenance (Buckley and Turner, 2018), while occludin interacts directly with Caudins and actin in (Müller et al., 2005).

Our results showed that BJO rebounded 5-FU-induced decrease of these tight junction proteins, confirming that BJO could improve mucosal barrier function. When high permeability occurrs in the intestine during intestinal mucositis, DAO penetrates across the intestinal barrier and enters the bloodstream (Li et al., 2018; Jiang et al., 2020). Hence, the decrease of intestinal DAO level reflects the impairment of mucosa integrity (Miyoshi et al., 2015). Our observations that intestinal DAO level was elevated by BJO, provide further evidence that BJO could restore tight junction damage in CIM.

To investigate the biological mechanisms of the protective potential of BJO in CIM, we studied Nrf2/HO-1 pathway that are critical in the regulation of oxidative stress (Zorov et al., 2014). The enhancement of Nrf2 nuclear transportation and the increased expression of Nrf2 target gene HO-1 by BJO in CIM mice implied that the intestinal protective effects of BJO are probably attributable to activation of Nrf2. Indeed, the Nrf2/HO-1 signaling pathway is involved in orchestrated regulation of oxidative and inflammatory stress (Vasileva et al., 2020), as well as in the function of epithelial tight junction (Lau et al., 2015; Liu et al., 2017). Thus, the activation of Nrf2/HO-1 might help to provide one possible explanation for the antioxidant and improving anti-inflammatory status of BJO in the small intestine, and the restoration of mucosal barrier function by BJO. Collectively, the protective effects of BJO in CIM are most likely attributable to activation of Nrf2/HO-1.

As stated above, the present study demonstrated that BJO could ameliorate inflammation and diarrhea in 5-FU-induced CIM. Therefore, we believe that appropriate dose of BJO could be used as a potential novel therapeutic strategy for the prevention against intestinal mucositis event. Moreover, a previous study showed that the traditional Chinese medicine relieves mucositis corresponds with an amended intestinal flora. These components either directly improve the gut microbiome diversity and composition or have been metabolized by gut bacteria before being absorbed into the body. Therefore, we speculate that the intestinal flora might partially contribute the ability of BJO to ameliorate the intestinal mucositis induced by 5-FU. This is a hypothesis and needs further investigation for confirmation.

## 5 Conclusion

The present study identified that BJO has the potential to be used as a therapeutic agent for improved management of CIM. BJO significantly improved CIM syndrome in 5-FU-treated mice. The protective effects of BJO against CIM were attributed to the following respects: (1) relieving oxidative stress; (2) inhibiting intestinal inflammation; (3) preventing intestinal epithelial cell apoptosis but enhancing cell-proliferation capability of enterocyte; and (4) improving mucosal barrier function by upregulation of tight junction proteins. The possible mechanisms underlying the protective effects of BJO may be *via* activation of Nrf2/HO-1.

## Data Availability

The original contributions presented in the study are included in the article/supplementary material, further inquiries can be directed to the corresponding authors.

## Abbreviations

5-FU, 5-Fluorouracil; BJO, *Brucea javanica* oil; CIM, chemotherapeutic intestinal mucosal injury; Nrf2, Nuclear factor E2-related factor 2; HO-1, Heme Oxygenase-1; SOD, superoxide dismutase; MDA, malondialdehyde; COX-2, cyclooxygenase-2; CXCL1, CXC chemokine ligand 1; CXCL2, CXC chemokine ligand 2; Bax, BCL2-associated X protein; Bcl-2, B-cell lymphoma-2; Caspase-3, Cysteinyl aspartate specific proteinase-3; PCNA, proliferating cell nuclear antigen; ZO-1, Zonula occludens-1; ELISA, enzyme-linked-immunosorbent serologic assay; IL-1β, Interleukin-1β; IL-4, Interleukin-4; IL-6, Interleukin-6; TNF-α, tumor necrosis factor-α; iNOS, Inducible nitric oxide synthase; DAO, Diamine oxidase; GAPDH, glyceraldehyde-3-phosphate dehydrogenase; LO, loperamide; DAB, diaminobenzidine method; ROS, reactive oxygen species; ANOVA, one-way analysis of variance; PVDF, polyvinylidene fluoride.
